# Effect of 0.12% chlorhexidine in reducing microorganisms found in aerosol used for dental prophylaxis of patients submitted to fixed orthodontic treatment

**DOI:** 10.1590/2176-9451.19.3.095-101.oar

**Published:** 2014

**Authors:** Isis Rodrigues Menezes dos Santos, Ana Cristina Azevedo Moreira, Myrela Galvão Cardoso Costa, Marcelo de Castellucci e Barbosa

**Affiliations:** 1 Degree in Dentistry, Federal University of Sergipe, UFS.; 2 Adjunct professor, Department of Buccal Microbiology, Federal University of Bahia, UFBA.; 3 Professor, Postgraduate program, Federal University of Bahia, UFBA.

**Keywords:** Aerosol propellant, Effects of air contamination, Chlorhexidine

## Abstract

**Objective:**

This study aimed at assessing, *in vivo*, whether the prior use of 0.12% chlorhexidine as mouthwash would decrease air contamination caused by aerosolized sodium bicarbonate during dental prophylaxis. The study was conducted with 23 patients aged between 10 and 40 years old who were randomly selected and undergoing fixed orthodontic treatment.

**Methods:**

The study was divided into two phases (T_1_ and T_2_) with a 30-day interval in between. In both phases, dental prophylaxis was performed with aerosolized sodium bicarbonate jetted to the upper and lower arches for 4 minutes. In T_1_, 10 minutes before the prophylaxis procedure, the participants used distilled water as mouthwash for one minute; whereas in T_2_, mouthwash was performed with 0.12% chlorhexidine. Microbial samples were collected in BHI agar plates for microbiological analysis. Two dishes were positioned on the clinician (10 cm from the mouth) and a third one at 15 cm from the patient's mouth. The samples were incubated for 48 hours at 37°C. Results were expressed in colony-forming units (CFU).

**Results:**

Statistical analysis carried out by means of Student's t test, as well as Wilconxon and Kruskal-Wallis tests revealed that the prior use of 0.12% chlorhexidine as mouthwash significantly reduced CFU in the three positions studied (P < 0.001).

**Conclusion:**

The prior use of 0.12% chlorhexidine as mouthwash significantly reduced contamination caused by aerosolized sodium bicarbonate during dental prophylaxis in the orthodontic clinic.

## Abstract (pt)

**Objetivo:**

avaliar, *in vivo*, se a utilização prévia do bochecho com clorexidina a 0,12% diminui a contaminação do ar gerada pelo jato de bicarbonato de sódio durante a profilaxia dentária. O estudo foi realizado com 23 pacientes, na faixa etária entre 10 e 40 anos, escolhidos aleatoriamente, que faziam uso de aparelho ortodôntico fixo.

**Métodos:**

o estudo foi dividido em duas fases (T_1_ e T_2_), com intervalo de 30 dias entre elas. Em ambas, foi realizada profilaxia dentária com jato de bicarbonato de sódio na arcada superior e inferior, durante quatro minutos. Em T_1_, 10 minutos antes do procedimento, os participantes realizaram bochecho com água destilada durante um minuto, e, em T_2_, o bochecho foi realizado com clorexidina a 0,12%. Amostras dos microrganismos foram coletadas em placas de ágar BHI para análise microbiológica, sendo duas placas posicionadas no profissional (a 10cm da boca) e uma terceira a 15cm da boca do paciente. Após a coleta, as placas foram incubadas por 48 horas a 37°C. O resultado foi expresso em número de unidades formadoras de colônias (UFC).

**Resultados:**

após análise estatística utilizando teste *t* de Student, teste de Wilconxon e teste de Kruskal-Wallis, observou-se que o bochecho prévio com clorexidina a 0,12% reduziu significativamente a média de UFC nas três posições estudadas (p < 0,001).

**Conclusão:**

os resultados permitem concluir que o bochecho prévio com clorexidina a 0,12% proporcionou uma redução estatisticamente significativa na contaminação gerada por meio do jato de bicarbonato de sódio durante a profilaxia dentária na clínica ortodôntica.

## INTRODUCTION

Cross infection control and biosecurity issues are crucial to the dental practice. Healthcare professionals and patients are often subjected to several risks, among which is cross infection.^1^ Reducing it is a major challenge for dentists, researchers and microbiologists.^2^

In some cases, microorganisms overcome the security measures adopted, thus putting patients and professionals at risk. This often occurs as a result of professional negligence with regard to biosecurity, which intensifies the cross infection cycle in the dental office.^3^ The orthodontic practice differs from other dental specialties by the volume of patients assisted per day, which increases the chances of cross infection.^4 ^Additionally, orthodontic treatment with fixed appliances increases biofilm accumulation.^5,6^

Given the negative effects of plaque accumulation during orthodontic treatment, orthodontists are constantly searching for new techniques and material that benefit and protect both patients and clinicians.^7,8^

The use of antimicrobial agents can help to maintain the integrity of tooth structure.^5^ Chlorhexidine is a chemical agent with antimicrobial properties capable of inhibiting bacterial growth and reducing the number of these microorganisms in the oral cavity, including *Streptococcus* associated with the development of caries.^2,9^

Aerosol particles may contain viruses, such as those of the acquired immunodeficiency syndrome (AIDS) and hepatitis B (HBV), which can penetrate through the clinician's, assistant's and patient's respiratory tract and conjunctiva membranes.^6,10^

For this reason, it is essential that clinicians and assistants cooperate to avoid cross contamination as a result of the use of aerosol equipment by means of which microorganisms can be introduced and spread within one meter around the operative field.^11^

The aim of this *in vivo* study was to assess whether the prior use of 0.12% chlorhexidine as mouthwash would decrease contamination caused by aerosolized sodium bicarbonate during dental prophylaxis of patients undergoing fixed orthodontic treatment.

## MATERIAL AND METHODS

This quantitative longitudinal study was conducted with patients undergoing orthodontic treatment carried out by the Postgraduate Program in Orthodontics, School of Dentistry, Federal University of Bahia (UFBA).

All participants were strictly treated in accordance with Resolution 196/96 issued by the Brazilian National Health Council (CNS). The research was approved by the UFBA Institutional Review Board through consolidated opinion Nº. 171.801 and registration in SISNEP, CAAE 03798312.2.0000.5024.

Sample size calculation was performed to detect a difference of 20% in relation to the initial data.^12^ According to data provided by the G*Power program (version 3.3, G*Power Software, Inc. 1 Mannheim, Germany), 22 individuals were necessary for each group.

### Sample characterization

In selecting the sample, the following inclusion criteria were applied:

1. A minimum of five teeth in each quadrant of the upper and lower arches;
2. Absence of systemic diseases;
3. No previous use of antibiotics or antiseptic mouthwash in the last 30 days;
4. No previous professional prophylaxis in the last 30 days.

Initially, a total of 25 patients were selected for this study. After applying the inclusion criteria, the number was reduced to 23, males and females aged between 10 and 40 years, randomly chosen and who were undergoing fixed orthodontic treatment.

To maintain the same treatment conditions and avoid interference in the results, the study was conducted in the same booth of the Postgraduate clinic. Before each procedure, the dental equipment was decontaminated with 2% chlorhexidine and alcohol 70%.^13^ The handpiece used to jet the aerosolized sodium bicarbonate was sterilized in an autoclave, and the water used for dental prophylaxis was distilled.

The research was conducted in two phases with a 30-day interval in between. The researcher used the following personal protective equipment: glove, mask, cap, goggles and lab coat; and followed the criteria of ideal biosecurity.^13^

**Figure 1:**
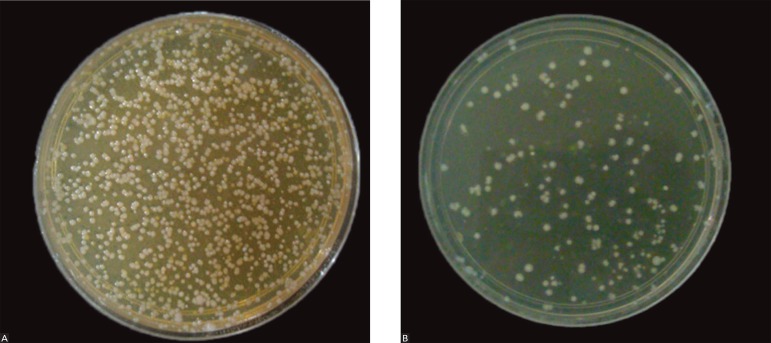
Dental prophylaxis and sample collection for microbiological analysis with dishes positioned at P1, P2 and P3.

Samples were collected in a dish containing 25 mL of BHI agar (Eximlab Commercial Equipment Laboratory LTD - Paraná - Brazil), placed onto the clinician's face (forehead area) (taped to a skullcap) and identified as P1. Another dish containing the same medium was positioned 10 cm from the clinician's mouth (vertical downward direction) and identified as P2. A third dish also containing BHI agar was placed over the patient's thoracic region, 15 cm from the oral cavity and identified as P3 (Fig 1).The dishes were made of 90 mm ​​x 15 mm, sterile, smooth Petri plastic (J. Prolab - Paraná - Brazil). The culture medium used was BHI agar. To avoid contamination, the Petri dishes containing 25 mL of BHI agar were exposed on the auxiliary table before procedure onset.

**Figure 2:**
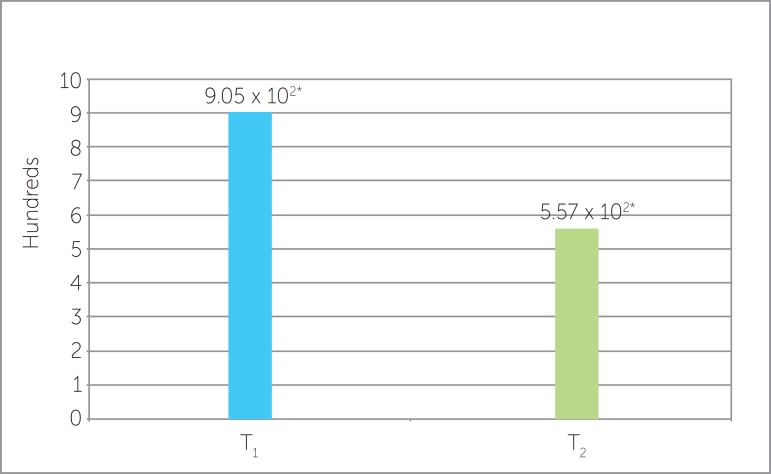
CFU means generated in dental prophylaxis with sodium bicarbonate spray distributed in T1 and T2 (p

Jet hand I sodium bicarbonate jet (Gnatus dental medical equipment LTD - São Paulo - Brazil) was used for dental prophylaxis (Fig 2).

### First phase (T_1_)

The patient rinsed the mouth with 15 mL of distilled water for one minute ten minutes before prophylaxis. Dental prophylaxis was performed for 4 minutes in the upper and lower quadrants of all subjects with sodium bicarbonate jet of which container was filled with distilled water.

### Second phase (T_2_)

After 30 days, all patients were subjected to a new prophylaxis procedure following the same aforementioned standards. However, mouthwash was performed ten minutes before prophylaxis with 15 mL of 0.12 % digluconate chlorhexidine for one minute.

### Microbiological evaluation

After sample collection, the dishes were identified and incubated under aerobic conditions at 37°C for 48 hours. After incubation, the total count of colonies was carried out for each dish using a colony counter model EC 550A (PHOENIX - São Paulo - Brazil). Results were expressed in CFU (Colony Forming Units).

### Statistical analysis

Data were tabulated in Excel spreadsheet for Windows 2010 and analyzed in GraphPad Prism (version 5.0, GraphPad Software. Inc., San Diego, CA, USA). Shapiro-Wilk test was used to assess sample normality. Student's t test was used for data with normal distribution (comparison of two means of P3). As for data with non normal distribution, Wilconxon test (comparison of two means of P1; comparison of two means of P2; and comparison of both overall means) and Kruskal Wallis (comparison of three positions in T_1 _and comparison of three positions in T_2_) were used. Significance level was set at P < 0.05 for all analyses.

## RESULTS

Assessment carried out in T_1_ and T_2_ for the dishes previously exposed on the auxiliary table before prophylaxis revealed no significant growth of CFU, with an average of two Colony Forming Units per dish.

Figure 1 shows a BHI agar dish, positioned at P1, where mesophilic bacteria colonies developed during prophylaxis with bicarbonate jet and subsequent incubation.

### Comparison between T_1_ and T_2_

Comparison of CFU means between T_1_ and T_2_ (mouthwash with distilled water and mouthwash with 0.12 % chlorhexidine) at P1, P2 and P3, revealed statistically significant differences (P < 0.001). In T_1,_ the mean of CFU was 9.05 x 10^2^, while in T_2_ it was 5.57 x 10^2^ during dental prophylaxis (Fig 2).

**Figure 3:**
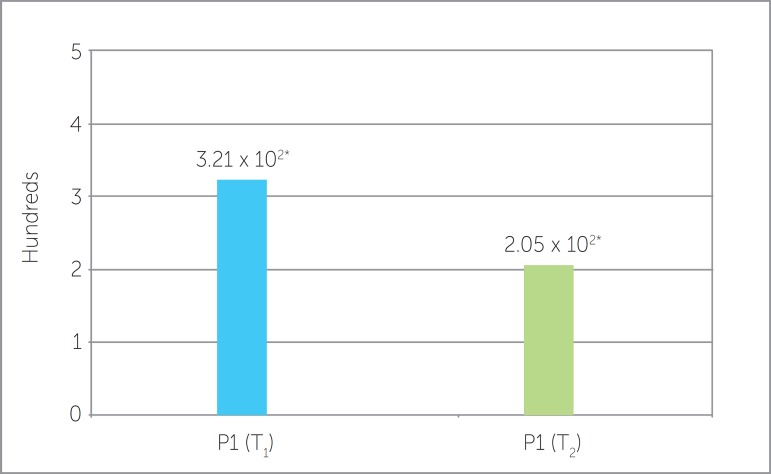
Comparison of CFU means at P1, in T1 and T2. (p=0.0074*). Standard deviation P1 (T1) = 274.79 and P1 (T2) = 174.34.

Comparison of dishes positioned at P1 in both T_1_ and T_2_ phases also revealed statistically significant differences (P = 0.0074), as shown in Fig 3. CFU means of 3.21 x 10^2^ were obtained in the first phase, whereas CFU means of 2.05 x 10^2^ were found after mouthwash with 0.12 % chlorhexidine.

**Figure 4:**
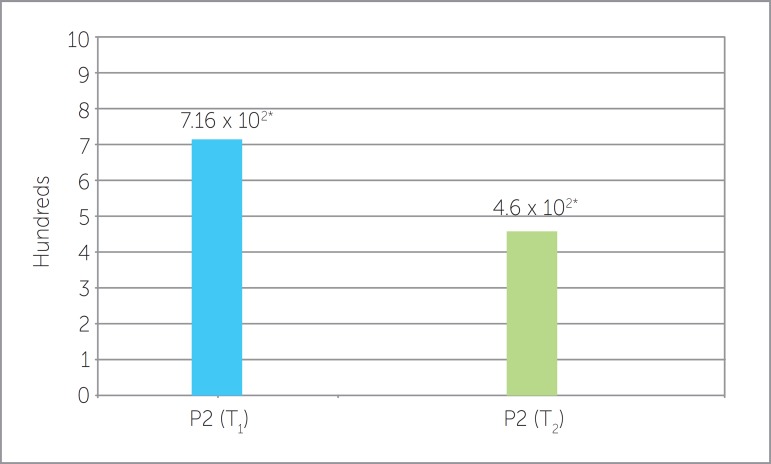
Comparison of CFU means at P2, in T1 and T2 (p=0.0051*). Standard deviation P2 (T1) = 351.03 and P2 (T2) = 226.76.

Comparison of dishes positioned at P2 in phases T_1_ and T_2_ also revealed a statistically significant reduction in CFU mean (P = 0.0051) (Fig 4).

**Figure 5:**
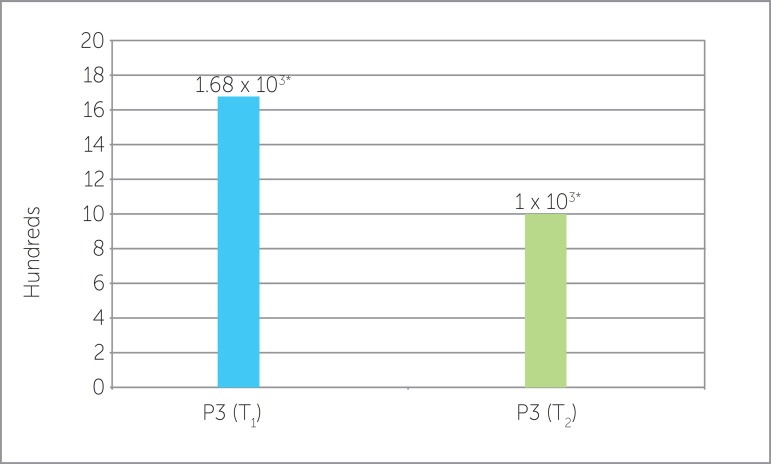
Comparison of CFU means at P3, in T1 and T2 (p=0.0035*). Standard deviation P3 (T1) = 953.80 and P3 (T2) = 554.93.

The dishes positioned in the patient (P3) revealed significant difference between T_1_ and T_2_ (P = 0.0035) (Fig 5).

### Comparison among P1, P2 and P3

**Figure 6:**
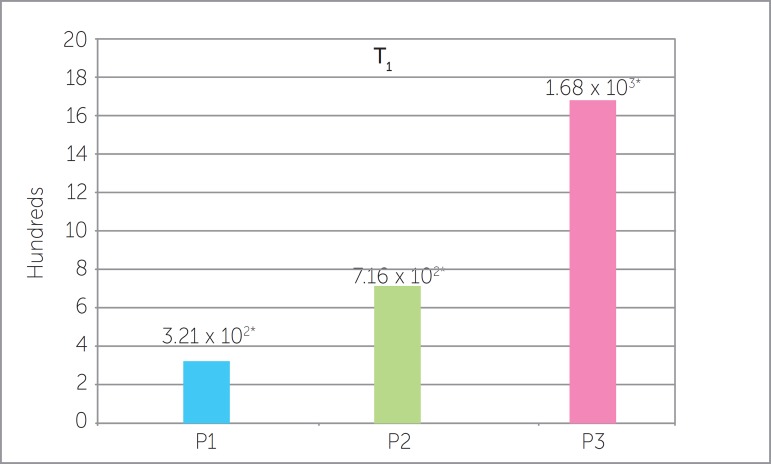
Comparison of CFU means at P1, P2 and P3, in T1 (p<0.05*).

**Figure 7:**
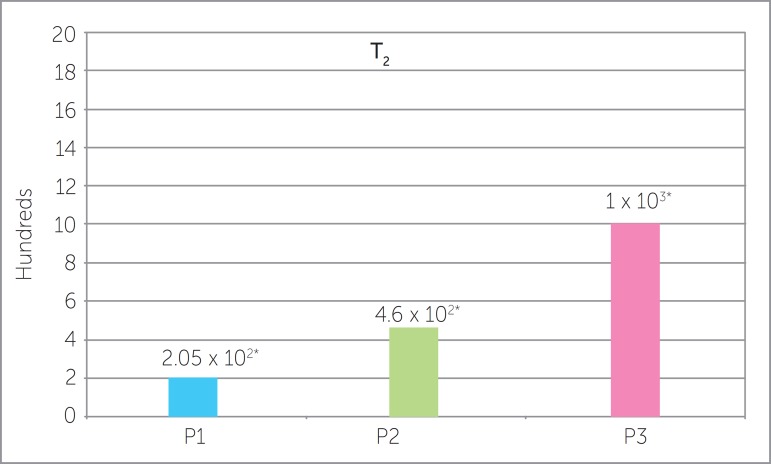
Comparison of CFU means at P1, P2 and P3, in T2 (p<0.05*).

Figures 6 and 7 compared the means of dishes positioned at P1, P2 and P3 in T_1_ and T_2_, respectively. The dishes positioned at P1, P2 and P3 showed statistically significant differences in both phases, whereas the dish positioned in the patient (P3) showed higher means in comparison to that positioned in the clinician. In the clinician, P2 showed higher CFU means than P1 in both steps.

## DISCUSSION

Given the possible changes in patient's oral microbiota and the increased risk of contamination involved during orthodontic treatment, it is essential that preventive methods be employed for all patients.^14^ The type, frequency and amount of measures adopted to prevent and maintain oral health will depend on the individual characteristics of the clinician and the patient.

Dental prophylaxis has proved to be one of the most important preventive methods.^15^ Studies have shown that the technique of prophylaxis carried out with sodium bicarbonate jet is effective in removing biofilm from all tooth surfaces, as well as from fissures and fossas. It is recommended for patients undergoing orthodontic treatment.^16^ In comparison to the rubber cup and pumice techniques, prophylaxis with sodium bicarbonate jet is considered the most advantageous technique for orthodontic patients. In spite of increasing the aerosol formed, the technique increases biofilm removal, reduces operative time and prevents heat release during the procedure.^17,18^ For these reasons, sodium bicarbonate jet was chosen for collection and evaluation of the aerosol method in this research.

Another method employed to prevent cross contamination is the use of antimicrobial agents. Chlorhexidine is considered the gold standard in comparison with other substances used to interfere in biofilm formation.^9,19,20^ It was most commonly used twice a day as a 10 mL, 0.2% mouthwash solution.^21^

However, Keijser et al^22^ and Quirynen et al^23^ compared the use of 0.12 % and 0.2% chlorhexidine solutions and found that both concentrations decreased contamination and, as a consequence, provided antimicrobial control.

Additionally, other studies have shown that decreasing the concentration and increasing the volume of the solution practically provides the same amount of drug with similar antiplaque effectiveness, but reduced side effects.^22^ Thus, mouthwash with 15 mL of chlorhexidine at 0.12% has been used on a large scale. At these concentrations, the recommended time for rinsing is one minute.^20^

This study assessd and compared the clinical efficacy of 0.12 % chlorhexidine solution, used as previous mouthwash for one minute, in reducing contamination in an orthodontic environment. The results of this study reveal that 0.12 % chlorhexidine used as mouthwash ten minutes before dental prophylaxis significantly reduced the CFU number of bacteria present in aerosol produced during prophylaxis of orthodontic patients. This result corroborates the study conducted by Gonçalves et al^12^ which assessed, *in vivo*, the contamination generated by aerosol produced by a low-speed handpiece used for dental prophylaxis of non-orthodontic patients. They found a statistically significant reduction in contamination when 0.12 % chlorhexidine was used before the procedure. The additional use of chlorhexidine as a mouthwash 10 minutes before the orthodontic procedure proves to be a favorable alternative to reduce cross-contamination during orthodontic treatment, especially in patients who have poor oral hygiene.

Toroglu et al^24^ also conducted a study to assess the efficacy of mouth rinse with 0.12 % chlorhexidine before removing, by means of a handpiece, the excess adhesive material and resin of orthodontic patients. They found that contamination was not significantly reduced, which does not corroborate the present research. The study developed by Toroglu et al^24^ did not inform the waiting time between the use of 0.12% chlorhexidine and the performance of the procedure.

Chlorhexidine remains active in the mouth where it is slowly released.^25^ In the present study, the waiting time of 10 minutes between the mouthwash with 0.12 % chlorhexidine and the prophylaxis procedure proved to be a favorable alternative to reduce cross contamination during orthodontic treatment, especially in patients with poor oral hygiene.

The study conducted by Logothetis and Martinez Welles^26^ compared the use of distilled water and 0.12% chlorhexidine as mouthwash solutions employed 30 minutes before dental prophylaxis to reduce contamination. Their results showed no significant differences, which does not corroborate the present study.

The additional use of an antimicrobial agent such as chlorhexidine may be more effective than mechanical rinsing with distilled water, only. The cationic molecule of chlorhexidine in an oral environment is quickly attracted by the negative charges of bacterial cell surface, which characterizes the bacteriostatic and bactericidal properties of chlorhexidine. Both characteristics are directly related to the concentration of the product.^2,20,22,23^ Using low-speed handpiece for dental prophylaxis, Gonçalves et al^12^ found an average of 37.2 CFU when comparing prophylaxis with and without previous mouthwash with 0.12 % chlorhexidine.

Studies assessing the amount of aerosol produced by sodium bicarbonate jet used as a method of dental prophylaxis revealed a significant increase of CFU when air/water spray was compared with low-speed traditional prophylaxis.^27,28^

The results yielded by this study revealed high CFU mean (7.31 x 10^2^ CFU). This can be explained by the type of patient selected for the study: Patients undergoing orthodontic treatment tend to have increased biofilm and, as a consequence, increased number of bacteria in the oral cavity. Additionally, it can also be explained by the use of bicarbonate spray which further increases the level of aerosol.

There is a wide variety of studies about the contamination of dental offices, particularly with regard to people directly involved with care.^9,12^ Gonçalves et al^12^ conducted a study in which they used agar dishes attached to the face of clinicians and assistants, as well as on patient's chest. Results revealed that contamination detected in the clinician resembles that found in the patient's chest.

In the present study, the areas of dish setting were selected so as to include areas where contamination is evident, thus ensuring the reach of the aerosol and the functionality of physical barriers in cross infection control.

CFU means at P3 had significant differences in comparison to P1 and P2 in both phases. The greater the proximity of the working area, the greater the bacterial dissemination.^1^ The study by Toroğlu et al^6^ assessed the amount of contamination caused by aerosol during removal with handpiece of adhesive material and resin excesses in orthodontic patients. Their results revealed that clinicians should worry about protecting the face, as well as areas of the neck and arms, given that these areas can be easily contaminated by aerosol spray.^6,24^

## CONCLUSION

Based on the results of this study it is reasonable to conclude that mouthwash with 0.12 % chlorhexidine performed before prophylaxis procedures significantly reduced contamination caused by aerosol of sodium bicarbonate spray used during dental prophylaxis in patients undergoing orthodontic treatment (P < 0.001).
